# Novel PEEK Copolymer Synthesis and Biosafety—I: Cytotoxicity Evaluation for Clinical Application

**DOI:** 10.3390/polym11111803

**Published:** 2019-11-02

**Authors:** Joon Woo Chon, Xin Yang, Seung Mook Lee, Young Jun Kim, In Sung Jeon, Jae Young Jho, Dong June Chung

**Affiliations:** 1 Department of Polymer Science & Engineering, Sungkyunkwan University, Suwon 16419, Korea; chonjoon@skku.edu; 2 School of Chemical Engineering, Sungkyunkwan University, Suwon 16419, Koreatmdanr1102@naver.com (S.M.L.); 3 School of Chemical and Biological Engineering, Seoul National University, Seoul 08826, Korea; ajeon88@gmail.com

**Keywords:** PEEK copolymer synthesis, PEEK composite, Spine cage application, In vitro biosafety

## Abstract

In this research, we synthesized novel polyetheretherketone (PEEK) copolymers and evaluated the biosafety and cytotoxicity of their composites for spinal cage applications in the orthopedic field. The PEEK copolymers and their composites were prepared through a solution polymerization method using diphenyl sulfone as a polymerization solvent. The composite of PEEK copolymer showed good mechanical properties similar to that of natural bone, and also showed good thermal characteristics for the processing of clinical use as spine cage. The results of an in vitro cytotoxicity test did not show any evidence of a toxic effect on the novel PEEK composite. On the basis of these cytotoxicity test results, the PEEK composite also proved its in vitro biosafety for application to an implantable spine cage.

## 1. Introduction

The spine plays two important and distinct roles. It provides a strong central axis for the appendicular skeleton and protects the spinal cord and roots of delicate nerves connected to the brain. Therefore, the artificial bone graft materials used in spinal cage development must have proper strength and stiffness, as well as the capability to bond to vertebrae. Metallic materials, such as titanium alloy and stainless steel, have been commonly used in spinal cages with competent mechanical and biologically inert properties [1,2]. Despite the outstanding benefits of metallic materials for spinal cage applications, several concerns have been raised due to the stress shielding effect, low biocompatibility, release of ionic effluence, magnetic image interference, and additional second surgery required for removal. Typically, the Young’s modulus of these alloys (titanium alloy: 80–125 GPa, magnesium alloy: 41–45 GPa) is shown to be higher than that of human bone (3–20 GPa). The mismatch of the Young’s moduli between metallic materials and the human bone could induce a stress shielding effect on the bone [3], which can lead to implant loosening, bone thickening, and chronic inflammation [4].

To overcome the several concerns associated with the use of metallic materials, polyaryletherketones (PAEKs), including the polyetheretherketone (PEEK) group, have been employed as biomaterials for spinal cages [5]. PEEK polymers have specific thermal processing conditions due to their crystal structures [6]. Therefore, a copolymerization method between various PEEKs was applied to fit the mechanical properties of PEEK polymers [7]. PEEK polymers find applications as high performance super engineering plastics. However, due to its high glass transition temperature (*T*_g_) and high melting point (*T*_m_) [8,9] the processing temperature of PEEK is very high. Several modifications were reported in order to reduce the processing temperature, such as lowering the *T*_m_ and softening the backbone of the PEEK [10,11,12]. In the present paper, P(E2–E4)K was synthesized using HQ, PD, and 4,4′-DFBP. As the reactant, PD has the structure of “-ether-phenyl-ether-phenyl-ether-phenyl-ether” which contains four ethers (E4: EEEE). Therefore, the introduction of PD into P(E2–E4)K greatly affected the distribution of the flexible and rigid segments on their molecule chains.

PEEK polymers were growing interest for their clinical application as a plate in fracture fixation due to their stiffness and similar Young‘s moduli to that of the human bone [13]. According to recent reports, their Young’s moduli can be fitted to closely match that of the human bone (3–20 GPa) by preparing a composite with a reinforcement treatment or modifying the chemical structure by increasing the molecular weight [14,15,16].

Regarding the mechanical strength of cortical bone, the flexural and compressive strength of human cortical bone have been reported to be in the range of 103–238 and 130–213 MPa, respectively [17,18]. Since the mechanical strength of PEEK is in the low range, PEEK should be further reinforced to fit in the high range in order to be applied for various patients. One of carbon fillers that is commonly used as a reinforcement for the spinal cage is carbon fiber (CF) because of its good mechanical properties, thermal resistance, wearability, and biocompatibility [19,20]. Another carbon filler that can be used is graphene oxide (GO), a promising filler for both mechanical and biological applications due to its high surface area and hydrophilic oxygen-rich functional groups on the surface [21]. Several studies show that the mechanical properties of various polymers were enhanced by adding GO but decreased after adding 1 wt % of GO or more due to the aggregation of GO nanosheets [22,23,24]. According to recent studies, a low concentration of GO has been confirmed to be non-toxic and biocompatible because of the oxygen-rich functional groups which enhance cell adhesion and spreading [25,26,27].

In this study, we aimed to verify the potential in vitro biocompatibility of various reinforced PEEK composites for applicability to spinal surgery. Further, to enhance mechanical properties, a PEEK composite which blended with novel PEEK copolymers and various reinforcements was developed. An in vitro analysis was performed using an MTT assay to confirm the cytotoxicity of the PEEK composites. A live/dead cell assay was used to visually determine the cell viability. The cell activity via the charge of phosphatase derived from the living cell membrane was detected by the alkaline phosphatase (ALP) assay.

## 2. Materials and Methods

### 2.1. Materials

Hydroquinone (HQ), sodium carbonate (Na_2_CO_3_), phosphate-buffered saline (PBS), penicillin-streptomycin, trypsin-EDTA, a Live/Dead Cell assay kit, and MTT-assay kit were purchased from Sigma-Aldrich Chem. Co. (St. Louis, MO, USA). Further, 4,4′-Difluorobenzophenone (4,4′-DFBP) and diphenyl sulfone (DPS) were purchased from TCI Co. Ltd. (Tokyo, Japan). Fetal bovine serum and Dulbecco’s modified Eagle medium (DMEM) were purchased from GE Healthcare Life Sciences (Logan, UT, USA). 4,4′-(1,4Phenylenebis(oxy)) diphenol (PD) was kindly provided by the Organosynthesis Lab., Dept. of Chemistry of Incheon National University (Incheon, Korea). The ALP assay kit was purchased from AnaSpec. Co. Inc. (Fremont, CA, USA). L-929 mouse fibroblast cells were obtained from the Korean Cell Line Bank Co. (Seoul, Korea).

P(E2–E4)K powder, graphene oxide powder (purity: >99 wt %, lateral dimension: ≥7 μm, thickness: 1.1~1.3 nm, LS-Chem, Ochang, Korea), and milled carbon fiber (PX 35 Milled Carbon Fibers, average length: 150 μm, average diameter: 7.2 μm, specific gravity: 1.81 g/cm^3^, Zoltek Co., St. Louis, MO, USA) were used for the preparation of composite samples using synthesized PEEK copolymers.

### 2.2. PEEK Polymerization and Composite Preparation

**Figure 1:**
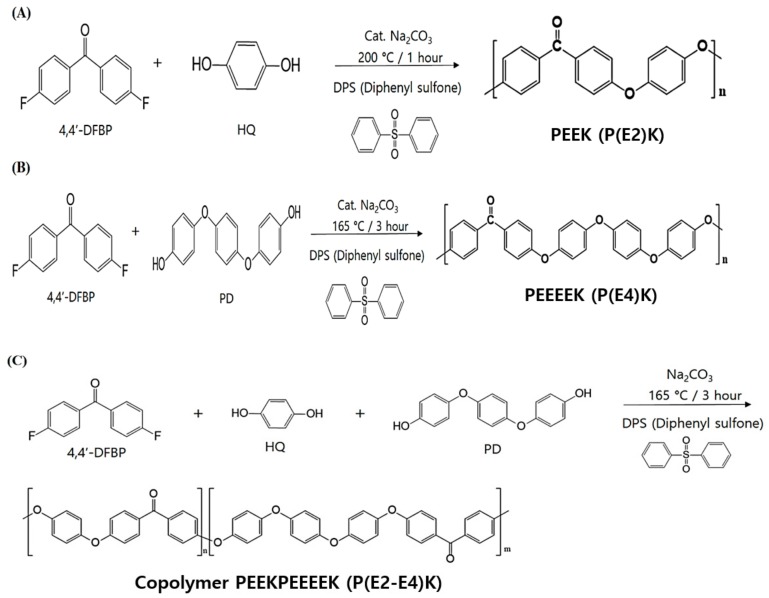
Chemical synthesis schemes of the PEEK polymers for controlling the number of ether groups in the repeating unit. (**A**) PEEK polymerization for P(E2)K, (**B**) PEEEEK polymerization for P(E4)K, and (**C**) the copolymerization of P(E2)K and P(E4)K to P(E2–E4)K.

Novel PEEK (P(E2)K and P(E4)K) and its copolymer (P(E2–E4)K) were polymerized by a solution polymerization method with various formulation for controlling the number of ether groups in the repeating unit. (Figure 1) After preparing PEEK and its copolymer, two kinds of reinforcements (CF: carbon fiber and GO: graphene oxide) were blended with the PEEK copolymers using suspension blending method (Table 1). The contents of GO were fixed to 0.5 wt % (see Supplementary Materials).

#### 2.2.1. Synthesis of Poly(ether ether ketone) P(E2)K

A mixture of hydroquinone (HQ, 10.00 mmol), 4,4′-difluorobenzophenone (4,4′-DFBP, 10.00 mmol), ground sodium carbonate (Na_2_CO_3_, 10.2 mmol), and diphenyl sulfone (DPS, 200 wt %) was purged by vacuum/nitrogen cycles using a Schlenk line. This mixture was stirred under nitrogen atmosphere and heated at 160 °C for 2 h, 250 °C for 2 h, at 320 °C for 1 h, successively. The obtained grey slurry was cooled to room temperature and grounded to a fine powder. The obtained product was washed in hot acetone (3 times) and hot DI water (3 times). Finally, the product was dried under high vacuum (ca. 0.05 mbar) at 140 °C for 12 h, to afford a grey solid.

#### 2.2.2. Synthesis of Poly(ether ether ether ether ketone) P(E4)K

A mixture of 4,4′-(1,4phenylenebis(oxy)) diphenol (PD, 10.00 mmol), 4,4′-DFBP (10.00 mmol), Na_2_CO_3_ (10.2 mmol), and DPS (200 wt %) was purged by vacuum/nitrogen cycles using a Schlenk line. This mixture was stirred under nitrogen atmosphere and heated successively at 160 °C for 2 h, at 235 °C for 2 h, and finally at 290 °C for 5 h. The obtained brown slurry was cooled to room temperature and grounded to a fine powder. The obtained product was washed with hot acetone (3 times) and hot DI water (3 times). Finally, the product was dried under high vacuum (ca. 0.05 mbar) at 140 °C for 12 h, to produce a brown solid.

#### 2.2.3. Synthesis of the Random Copolymer P(E2–E4)K

A mixture of HQ (9.00 mmol), PD (1.00 mmol), 4,4′-DFBP (10.00 mmol), Na_2_CO_3_ (10.2 mmol), and DPS (200 wt %) was purged by vacuum/nitrogen cycles using a Schlenk line. This mixture was stirred under nitrogen atmosphere and successively heated at 160 °C for 2 h, 240 °C for a further 2 h, and finally at 300 °C for 5 h. The obtained grey slurry was cooled to room temperature and ground to a fine powder. The obtained product was washed with hot acetone (3 times) and hot DI water (3 times). Finally, the product was dried under high vacuum (ca. 0.05 mbar) at 140 °C for 12 h, to produce a grey solid.

#### 2.2.4. Fabrication of the PEEK Composite

To prepare the blend powders, P(E2–E4)K powder and fillers were separately dispersed in a beaker containing ethanol, followed by ultrasonication for 30 min. Subsequently, each dispersed powder sample was mixed with another using a magnetic stirrer for 12 h, according to the composition provided in Table 1. The composite suspension was filtered and vacuum-dried at 60 °C for 24 h.

For an evaluation of the mechanical properties and bio-safety, the blended powder samples were molded into 10 × 10 × 4 mm^3^ plates using a mini-injection molding machine (Bautek Co., Uijeongbu-si, Korea) at processing temperatures of 390 °C, with a preset molding temperature of 190 °C. A flexural test specimen of 80 × 10 × 4 mm^3^ and compression test specimen of 10 × 10 × 4 mm^3^ were also prepared using the above procedure. To ensure a similar degree of crystallinity, all the samples were annealed at 220 °C for 4 h.

### 2.3. Characterization and Mechanical Properties of the Synthesized PEEK Polymers and Composites

Using an Ubbelohde viscometer (Xylem Inc., Rye Brook, New York, USA), the inherent viscosities (*η*_inh_*) of the synthesized PEEK polymers (including copolymer) solutions (dissolved in concentrated sulfuric acid (98%) with a concentration of 0.5 g/dL) were measured at 30 °C. To confirm the thermal properties, differential scanning calorimetric (DSC) was measured with polymer powder samples using a DSC Q20 (TA Instruments, New Castle, PA, USA) under nitrogen flow, with a heating rate of 10 °C/min (25 to 350 °C). Perkin Elmer TGA 7 thermal analyzer (Perkin Elmer, Boston, MA, USA) was used to characterize the thermogravimetric properties of synthesized PEEK polymers. In brief, 10 mg of sample was placed in a sample pan and test was performed under nitrogen flow, with a heating rate of 10 °C /min (50 to 800 °C). Fourier transform infrared spectroscopy (FT-IR) spectra were obtained using a Nicolet™ iSTM 50 (Thermo Fisher Scientific, Waltham, MA, USA). Spectra were recorded using a spectral width ranging from 500 to 2000 cm^−1^ and an accumulation of 32 scans.

The flexural strength and compressive strength were measured according to the ISO 178 and ISO 604 standards, respectively. A universal test machine (Lloyd LR10K, West Sussex, UK) with a load cell of 10 kN was used for the test. The cross-head speeds were 2 mm/min and 1 mm/min for flexural and compression testing, respectively. The average of five measurements was obtained from seven specimens for each test.

### 2.4. In vitro Cytotoxicity Test of the PEEK Composites

Every sample for in vitro cytotoxicity test was extracted for 72 h at 36.5 °C with 4 g of P(E2–E4)K composites in 20 ml PBS. The formulation ratios of the additives in the P(E2–E4)K composites samples are shown in Table 1. Every cell experiment (The MTT-assay and ALP-assay) showing the cell viability and cell activity were carried out according the ISO 10993–5 standard. All experiments were carried out 5 times. Briefly, L-929 mouse fibroblasts were seeded (1 × 10^5^ cell/mL) into each well of a tissue culture polystyrene dish (TCPS). The cell was incubated at 36.5 °C in a 5% CO_2_ environment with DMEM medium containing 10% FBS and 1% penicillin-streptomycin. For the assay, each extracted sample solution described above was added to the TCPS wells and incubated with the cells for 2 days.

For the MTT-assay, DMEM (100 μL) and MTT (25 μL) solution were added to each well after 1%~20% of diluted extracted solution (100 μL) and the mixture was incubated for 4 h under the same conditions. After additional incubation, the supernatant was removed from each well by aspiration. Next, DMSO (100 μL) and glycine buffer (12.5 μL) were sequentially added to each well. For the ALP-assay, the same cell cultivation process as the MTT-assay was performed. To determine the cell viability and activity, the ultraviolet-visible (UV-Vis) absorbance of the solutions in each well was measured at 570 nm for the MTT-assay and at 405 nm for the ALP-assay using a SpectraMax M5 microplate reader (Molecular Devices Korea LLC, Seoul, Korea). For the live/dead Cell assay, samples were prepared using the same extraction protocol as the MTT assay. The specific extracted concentration was harsher than MTT assay (100% extracts solution/diluted solution to 50% concentration of original extracts) [28]. Next, calcein-AM and propidium iodide (PI) were added to each well followed by incubation for 15 min at 36.5 °C in a 5 % CO_2_ atmosphere. Fluorescence images were captured and analyzed using a Nikon Eclipse Ti microscope (Nikon Instruments Inc., Tokyo, Japan).

## 3. Results and Discussion

### 3.1. Synthesis and Characterizations of P(E2)K, P(E4)K, and P(E2–E4)K

Aromatic poly(ether ketone)s and copolymers were synthesized by typical nucleophilic displacement reaction. As shown in Figure 1, P(E2)K was prepared by HQ and 4,4′-DFBP with a molar ratio of 1:1.P(E4)K was prepared with PD and 4,4′-DFBP. P(E2)K showed a Tm value that was too high to process with extrusion or an injection molding, and P(E4)K had a relatively low molecular weight that diminished the mechanical properties. Therefore, its copolymers were synthesized to address polymer’s disadvantages. P(E2–E4)K was synthesized using HQ, PD, and 4,4′-DFBP. These three polymers were prepared in diphenyl sulfone under similar conditions (Table 2) and their molecule chains all consisted of a flexible segment (-phenyl-ether-phenyl-) and rigid segment (-phenyl-ketone-phenyl-). The reactant, PD, has the structure of “-ether-phenyl-ether-phenyl-ether-phenyl-ether”, which contains four ethers (E4: EEEE). Therefore, the introduction of PD into P(E2–E4)K greatly affected the distribution of the flexible and rigid segments on their molecule chains.

In both cases, the obtained polymers were insoluble in a wide range of organic solvents, except strong Brønsted acids (e.g., concentrated H_2_SO_4_). Therefore, the inherent viscosities were determined by measuring the flow time of their solutions in concentrated sulfuric acid (H_2_SO_4_) at 30°C. These polymers showed inherent viscosity values from 0.63 to 1.43 dL/g. P(E4)K showed the lowest inherent viscosity value among all prepared polymers. We assumed that the introduction of ether groups reduced the thermal stability of P(E4)K. However, a considerable reduction in viscosity was not observed for P(E2–E4)K copolymers where PD feed was 10%. Since we were unable to find the exact values of Mark–Houwink constants of K and α. We could not estimate the Mv of these polymers. Here, we present the inherent viscosity values instead. Comparing with previously reported inherent viscosity values of PEEK [10,29], we are certain that high molecular weight PEEK homo and copolymers are prepared.

**Figure 2:**
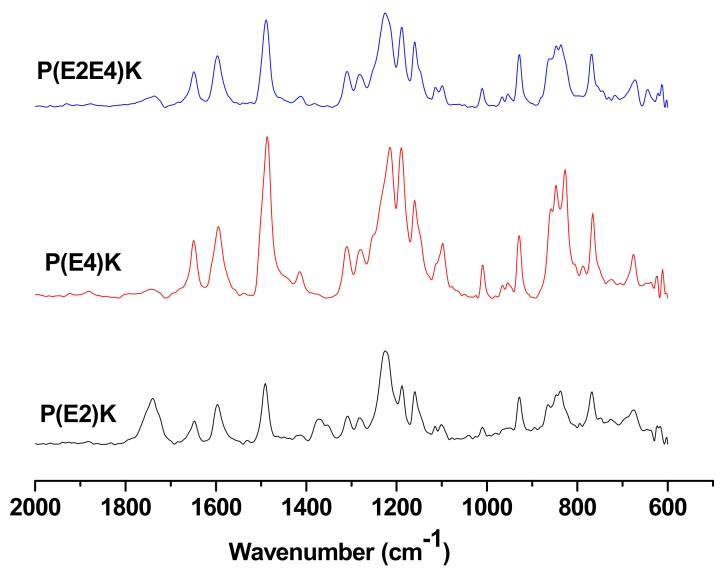
FT-IR spectra of the PEEK homo and copolymers. (Absorbance vs. Wavenumber).

The FTIR spectroscopy was further utilized to characterize the obtained polymers for estimating the copolymer composition. Figure 2 shows FTIR spectra of the PEEK homo and copolymers (absorbance vs. wavenumber). The bands at 1250, 1489 and 1650 cm^−1^ are due to C–O–C stretch, the aromatic C=C stretch and C=O stretch, respectively. The bands at 1489 and 1650 cm^−1^ are used as analytical bands since they are well separated from other bands. No specific differences between the three samples from the FTIR spectra because the functional groups of them are same. However, the phenyl group density of P(E4)K is higher than that of P(E2)K, which is used for estimating polymer composition. The ratio of peak area of C=O to aromatic C=C for P(E2)K, P(E4)K, and P(E2–E4)K were 2.43, 4.72, and 2.56. Considering the theoretical peak area ratio for P(E2–E4)K is 2.66, the observed peak area ratio of 2.56 for the copolymer can be accepted.

### 3.2. Thermal Properties of P(E2)K, P(E4)K, and P(E2–E4)K

**Figure 3:**
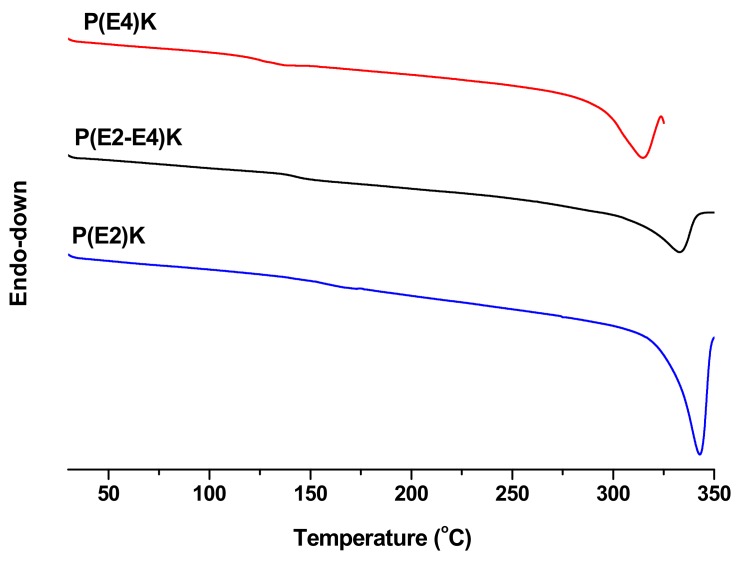
DSC thermograms of the PEEK homo and copolymers.

As shown in Figure 3, the *T*_m_ values of P(E2)K, P(E4)K, and P(E2–E4)K were investigated by DSC, and were 343, 315, and 333 °C respectively. As one of the most important parameters of high-performance plastics, the thermal property results indicate that introduction of a flexible segment decrease *T*_m_. The structure and property (*T*_m_ and *T*_g_) relationships for poly(ether ether ketone) are well known [30,31]. The incorporation of a flexible ether linkages (C–O–C) decreases *T*_m_ and *T*_g_ and the incorporation of ketone linkages increase *T*_m_ and *T*_g_.

**Figure 4:**
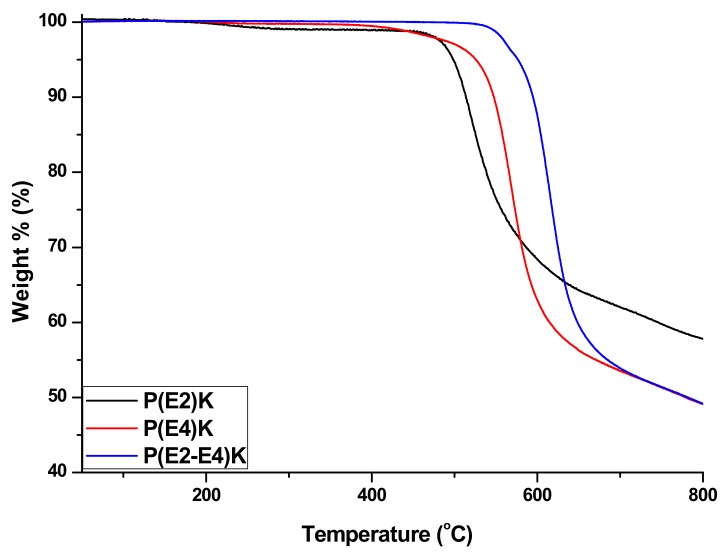
TGA curves of the PEEK homo and copolymers in a nitrogen atmosphere.

The TGA was performed under a nitrogen atmosphere. As shown in Figure 4, the three polymers exhibited excellent thermal stability. Their onset weight loss temperatures were all greater than 480 °C, particularly that of the P(E2–E4)K, probably owing to the relatively high molecular weight. These three polymers exhibited excellent properties based on the TGA and DSC test results which demonstrated that the sequence distributions did not affect their thermal properties.

### 3.3. Mechanical Properties

**Figure 5:**
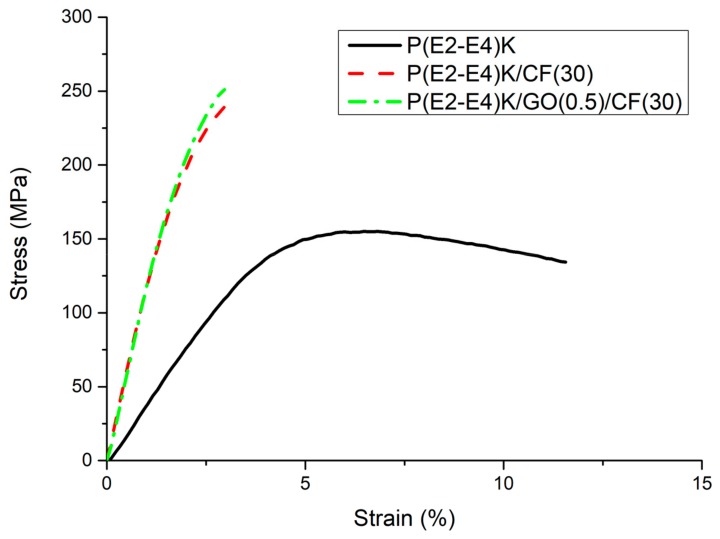
Flexural stress-strain curves of the P(E2–E4)K composite samples.

The flexural and compressive strength test results are shown in Table 3. The flexural strengths for the samples No. 1, No. 2, and No. 3 are 155, 240, and 250 MPa, respectively. The relationship between the flexural stress and strain in the composite samples is demonstrated in Figure 5. No. 2 and No. 3 exhibited higher yield stress and modulus but lower break energy compared to those of No. 1. The flexural strength of No. 2 and No. 3 were enhanced by 54.8% and 61.3%, respectively compared to that of No. 1 due to the high stiffness and modulus characteristics of the CFs [32]. The addition of GO increased the flexural strength by 10 MPa from No. 2 sample since GO plays a role in load transfer through strong interaction with copolymer matrix (see Supplementary Materials).

**Figure 6:**
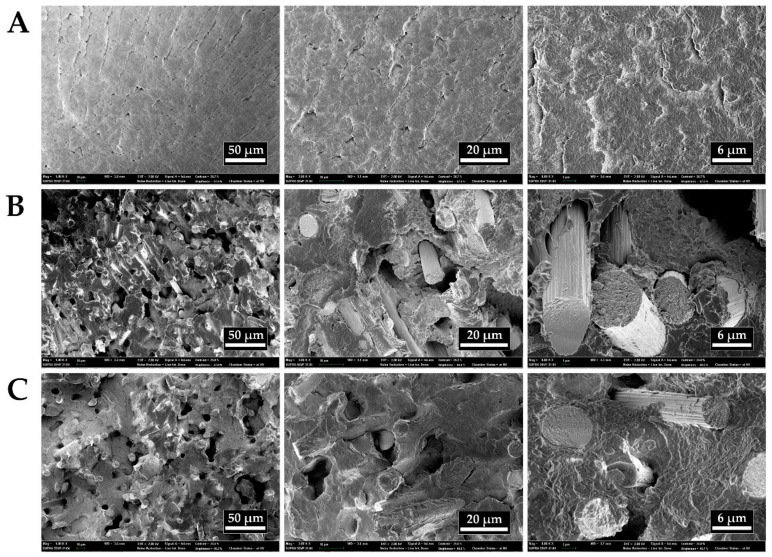
SEM images of flexural fracture surfaces of (**A**) No. 1, (**B**) No. 2, and (**C**) No. 3 samples.

The incorporation of GO improves the mechanical strength through two mechanisms: formation of π–π stacking interaction between the conjugated structure of GO and the benzene rings of copolymer, and hydrogen bonding between hydroxyl and carboxylic groups of GO and carbonyl group in the matrix [33,34]. The composite samples generally showed a sharp drop in strain after 2.8% due to the rigid characteristics of CF and GO fillers. This suggests a shift from ductile to brittle fracture behavior. As shown in Figure 6a, sample No. 1 showed smooth surface in conjunction with river patterns, indicating a typical ductile fracture behavior. When GO and CFs were added (Figure 6b–c), the fracture surface became much rougher with multilayer structure due to many interface debonding and pullout of CFs. Most of CFs in both No. 2 and No. 3 showed a smooth surface with little copolymer attached, indicating a weak interfacial adhesion between CF and copolymer matrix. This could be the main reason for the failure mode.

**Figure 7:**
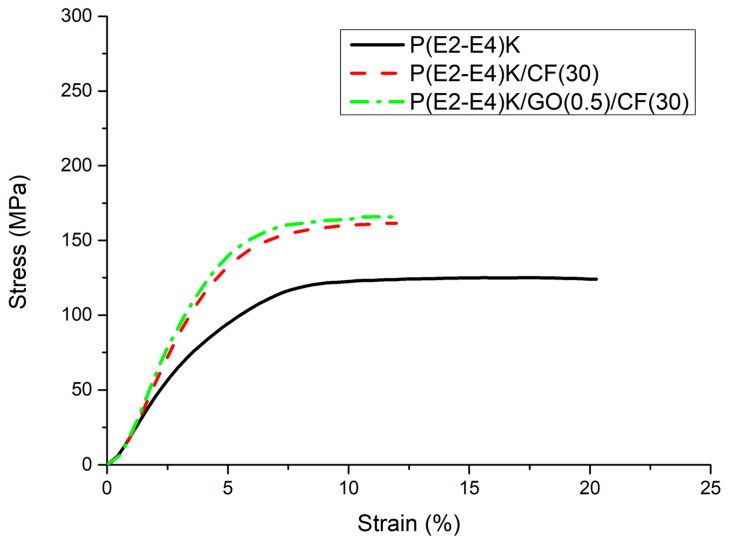
Compression stress-strain curves of the P(E2–E4)K composite samples.

The compressive strengths for No. 1, No. 2, and No. 3 in MPa were 125, 161, and 164, respectively, as shown in Table 3. Compared to the compressive strength of No. 1, those of No. 2 and No. 3 exhibited a sharp increase by 28.9% and 31.2%, respectively. As shown in Figure 7, the carbon fillers not only increased both yield strength and modulus, but also reduced the break energy, similar to the flexural strength test results. Furthermore, the compressive strength was slightly increased by 1.8% with the addition of GO. However, the improved compressive strength was primarily governed by the mechanical properties of the CFs rather than the properties of GO.

### 3.4. In Vitro Cytotoxicity Test of the PEEK Composites

**Figure 8:**
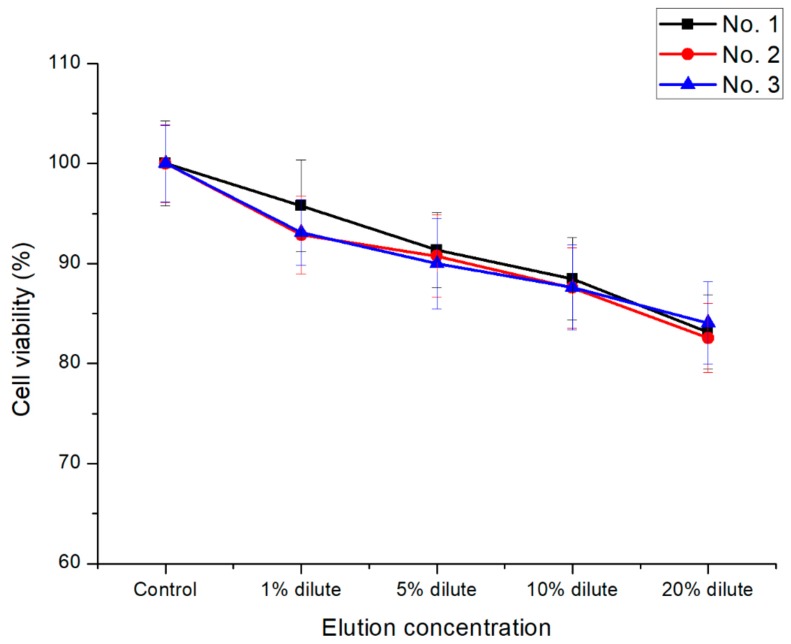
Cell viability results of the P(E2–E4)K composites via the MTT assay. Statistical analysis was conducted by one-way analysis of variance (ANOVA) and Turkey’s test; *p* < 0.05.

**Figure 9:**
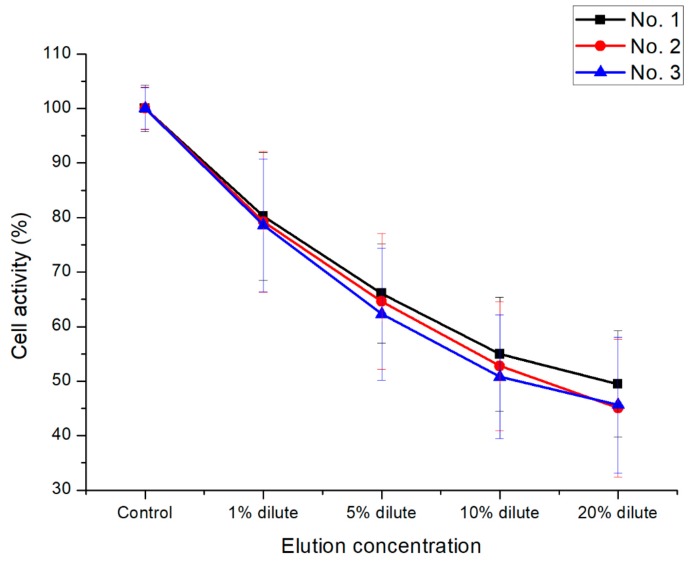
Cell activity results of the P(E2–E4)K composites via the ALP assay. Statistical analysis was conducted by one-way analysis of variance (ANOVA) and Turkey’s test; *p* < 0.05.

**Figure 10:**
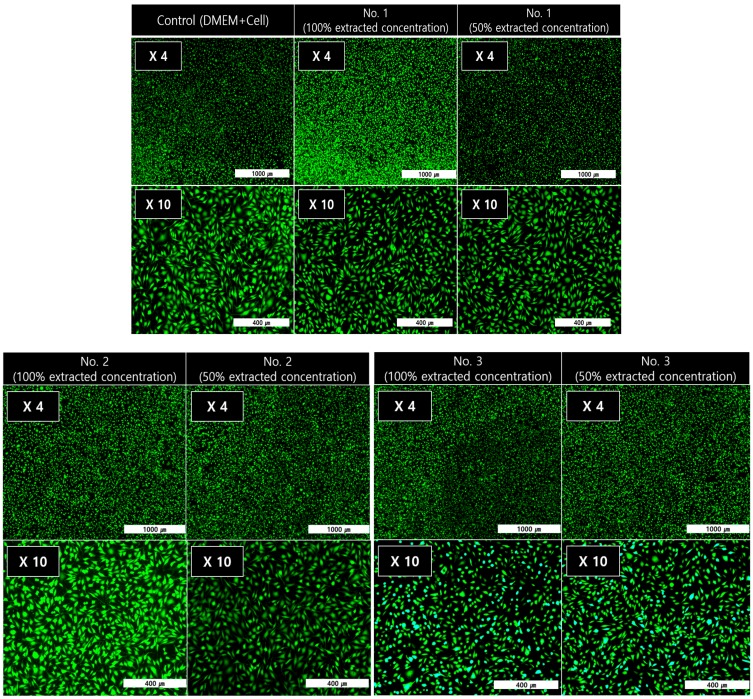
Images of cell viability via Live/Dead Cell-assay.

As an effective assessment tool for cytotoxicity, the reduction of mitochondria in living cells was evaluated to convert MTT tetrazolium (yellow) into MTT formazan (purple) to confirm the cell viability [35]. Variation of UV-Vis absorption forces at 570 nm varies in proportion to the reduction of MTT formazan. As shown in Figure 8, even if the concentration of the extract increased from 1% to 20% of the total volume of the medium, the cell survival capability at the equivalent advantage level (over 80%) was shown. As a result, all the samples showed non-toxic behavior in accordance with ISO 10993–5 standards. The ALP-assay kit was used to detect changes in the phosphate activity in living cell membranes to confirm the cell activity. As shown in Figure 9, P(E2–E4)K composite samples with various reinforcements showed an appropriate level of ALP activity compared to the neat sample (No. 1) case. Even when the concentration increased to 20% of the total media volume, the ALP activity was greater (or nearly identical) than that of the neat sample. As shown in the results of the live/dead cell assay (Figure 10), each sample had abundant live cells (green colored cells) without any dead cells (red colored cells), even when the concentration was greater than those in the other cytotoxicity tests. Furthermore, the equivalent cell population behavior was observed between the control group and each sample. These results mean that the PEEK composites containing various types of additives exerted no toxic effects and exhibited appropriate cell activity according to the in vitro cytotoxicity analysis data, per the ISO standard.

## 4. Conclusions

In this study, novel PEEK composites were synthesized for use in spinal cages as a prosthetic. (P(E2)K, P(E4)K), and their copolymer were synthesized via a typical nucleophilic displacement reaction, and a P(E2–E4)K composite with various reinforcements was prepared. The P(E2–E4)K composite showed enhanced thermal and mechanical properties compared to P(E2)K and P(E4)K for spinal cage applications. Based on the in vitro analysis, all the P(E2–E4)K composite samples were classified as non-toxic according to the ISO standard. Furthermore, each P(E2–E4)K composite sample had low cytotoxicity, even when its extracted volume increased to 20%. Based on the ALP assay, the P(E2–E4)K composite sample showed an appropriate positive value, indicating the capability of adjusting the PEEK composition for use in a spinal cage. These cytotoxicity data promote the applicability of the PEEK composites for orthopedic surgery.

## Figures and Tables

**Table 1:** Formulation ratios of the PEEK composites.

Sample No.	Samples Composition (wt %)
No. 1	P(E2-E4)K only
No. 2	P(E2-E4)K/CF(30)
No. 3	P(E2-E4)K/GO(0.5)/CF(30)

**Table 2:** Synthesis data for the preparation of aromatic PEEK homo and copolymers.

Polymer	Polymerization Solvent	Polymerization Temperature (°C)	Reaction Time (hr)	Yield (%)	*η*_inh_* (dL/g)
P(E2)K	Ph_2_SO_2_ (DPS)	160/250/320	2/2/1	96	1.20
P(E4)K	Ph_2_SO_2_ (DPS)	160/235/290	2/2/5	94	0.63
P(E2–E4)K	Ph_2_SO_2_ (DPS)	160/240/300	2/4/5	97	1.43

* Inherent viscosity measured in a ‘U’-tube viscometer for a 0.5 g/dL solutions in 96% H_2_SO_4_ at 30 °C.

**Table 3:** Flexural and compressive strength test results of the P(E2–E4)K composites.

Sample Name	Sample Composition (wt %)	Flexural Strength (MPa)	Compressive Strength (MPa)
No. 1	P(E2–E4)K	155 ± 3.6	125 ± 1.5
No. 2	P(E2–E4)K/CF(30)	240 ± 3.9	161 ± 2.9
No. 3	P(E2–E4)K/GO(0.5)/CF(30)	250 ± 4.0	164 ± 3.1

## Supplementary Materials

The following are available online at https://www.mdpi.com/2073-4360/11/11/1803/s1.
