# Supplementary material for: Novel PEEK Copolymer Synthesis and Biosafety—I: Cytotoxicity Evaluation for Clinical Application
Source: Polymers (Basel). 2019 Nov 2;11(11):1803. doi: 10.3390/polym11111803 (PMC6918363; doi:10.3390/polym11111803)
Supplement: Supplementary file 1 [file polymers-11-01803-s001.pdf]

Supporting Information

Novel PEEK Copolymer Synthesis and Biosafety – I :Cytotoxicity Evaluation for Clinical Application

Joon Woo Chon[1†], Xin Yang[2†], Seung Mook Lee[2], Young Jun Kim[*,2], In Sung Jeon[3†], Jae Young Jho[*,3], Dong June Chung[*,1]

1 Department of Polymer Science & Engineering, Sungkyunkwan University Suwon, 16419, Korea
2 School of Chemical Engineering, Sungkyunkwan University, Suwon, 16419, Korea
3 School of Chemical and Biological Engineering, Seoul National University, Seoul, 08826, Korea
* Correspondence: Dong June Chung(djchung@skku.edu); Young Jun Kim(youngkim@skku.edu); Jae Young Cho(jyjho@snu.ac.kr)

SI.1. Materials and composite preparation

PEEK powder (Victrex 450PF, Dict Co., Seoul, Korea) and graphene oxide (GO) powder (purity: >99 wt%, lateral dimension: ≥7 μm, thickness: 1.1~1.3 nm, LS-Chem, Ochang, Korea) were used for preparation of composite samples.

In order to investigate the effect of GO on the flexural strength of PEEK/GO composites, composites were prepared with GO contents of 0, 0.5, and 1 wt%. To prepare composite powders, PEEK powder and GO powder were separately dispersed in a beaker containing ethanol, followed by ultrasonication for 30 min. Then, each dispersed powders was mixed with another using a magnetic stirrer for 12 h, according to the composition given in **Table S1**. The composite suspension was filtered and vacuum-dried at 60 °C for 24 h.

Table S1. Formulation ratios of the PEEK composites.

Sample No.	Samples composition (wt%)
No. 1	PEEK only
No. 2	PEEK / GO(0.5)
No. 3	PEEK / GO(1)

For evaluation of flexural strength, blend powders were molded into 80 x 10 x 4 mm^3 plates using a mini-injection molding machine (Bautek Co., Uijeongbu-si, Korea) at processing temperature of 380 °C, with the pre-set mold temperature of 190 °C. For maintaining a similar degree of crystallinity, samples were annealed at 220 °C for 4 h.

A Universal Test Machine (Lloyd LR10K, West Sussex, UK) with a load cell of 10 kN was used to measure the flexural strength according to the ISO 178 standard. The cross-head speed was 2 mm/min. The average of five measurements was obtained from seven specimens.

SI.2. Mechanical properties of PEEK/GO composites

The flexural strength test results are shown in **Table S2**. The flexural strength for Samples No. 1, No. 2, and No. 3 was 154, 161, and 156 MPa, respectively. The relationship between flexural stress and strain in the composite samples is demonstrated in **Figure S1**. Compared with the neat PEEK (No. 1) sample, the flexural strength of No. 2 sample slightly increased by 4.5%. This is due to hydrogen

bonding and π-π stacking interaction. GO nanosheets, containing hydroxyl and carboxylic groups on the surface, can interact with carbonyl groups in PEEK matrix through hydrogen bonding. Moreover, the interfacial adhesion between GO and PEEK matrix can be enhanced through π-π stacking interaction due to the conjugated structure of GO and the benzene rings of PEEK. When the content of GO is further increased to 1 wt% (No. 3), both the flexural strength and the elongation at break are decreased. This result is similar with other GO reinforced polymer composites.[1-3] This reduction with increasing GO content can be due to the aggregation of GO nanosheets which can initiate the micro-sized cracks and decrease the yield strength as a result. As shown in **Figure S2**, the thickness of GO is drastically increased for No. 3 sample compared to No. 2 sample. The addition of GO content over 0.5 wt% leads to the aggregation of GO nanosheets, which can be found in voids on the fracture surfaces. Thereby, the flexural strength of PEEK/GO composite can be optimized at the GO content of 0.5 wt%.

Table S2. Flexural strength test result of the PEEK composites.

Sample No.	Sample composition (wt%)	Flexural strength (MPa)
No. 1	PEEK only	154±4.0
No. 2	PEEK / GO(0.5)	161±3.0
No. 3	PEEK / GO(1)	156±2.7

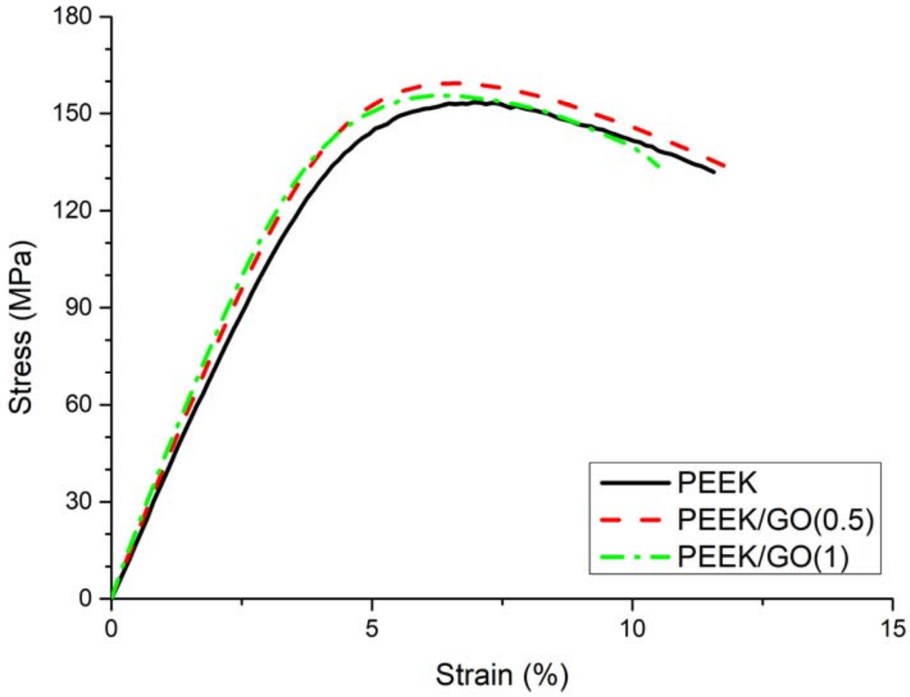

Figure S1. Flexural stress-strain curves of PEEK/GO composite samples.

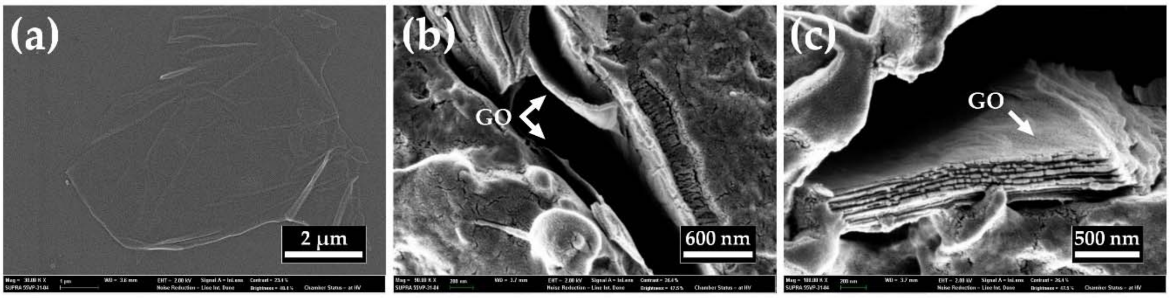

Figure S2. SEM images of (a) GO; and GO in the flexural fracture surface of (b) No. 2 and (c) No. 3 samples.

References

1. Pang, W.; Ni, Z.; Chen, G.; Huang, G.; Huang, H.; Zhao, Y. Mechanical and thermal properties of graphene oxide/ultrahigh molecular weight polyethylene nanocomposites. *RSC Adv.* **2015**, 5, 63063-63072
2. Wang, Y.; Shi, Z.; Fang, J.; Xu, H.; Yin, J. Graphene oxide/polybenzimidazole composites fabricated by a solvent-exchange method. *Carbon* **2011**, 49, 1199–1207
3. Chen, Y.; Qi, Y.; Tai, Z.; Yan, X.; Zhu, F.; Xue, Q. Preparation, mechanical properties and biocompatibility of graphene oxide/ultrahigh molecular weight polyethylene composites. *Eur Polym J.* **2012**, 48, 1026-1033
